# Effects of Tiaozhi Granule on Regulation of Autophagy Levels in HUVECs

**DOI:** 10.1155/2018/1765731

**Published:** 2018-07-12

**Authors:** Ying-Ying Wu, Chao Chen, Xiao Yu, Xiao-Dong Zhao, Rong-Qi Bao, Jia-Yu Yu, Guo-Xing Zhang, Jing-Wei Chen

**Affiliations:** ^1^Department of Internal Medicine, The Affiliated Suzhou Chinese Traditional Medicine Hospital, Nanjing University of Chinese Medicine, 18 Yang-Su Road, Suzhou 215003, China; ^2^Laboratory of Cancer Molecular Genetics, Medical College of Soochow University, 199 Ren-Ai Road, Dushu Lake Campus, Suzhou Industrial Park, Suzhou 215123, China; ^3^Department of Physiology, Medical College of Soochow University, 199 Ren-Ai Road, Dushu Lake Campus, Suzhou Industrial Park, Suzhou 215123, China

## Abstract

Sera from the rats with Tiaozhi granule treatment were collected. Human umbilical vein endothelial cells (HUVECs) were incubated with different dosage of sera with Tiaozhi granule for 48 hours. Rapamycin or angiotensin II was applied to activate autophagy in HUVECs with or without different dosages of sera of Tiaozhi granule. The mRNA expressions of Atg5, Atg7, Beclin-1, and mammal target of rapamycin (mTOR) were detected by real-time PCR. Autophagic flux markers (protein expression of LC3, Beclin-1, and p62) were examined by western blot analyses. The number of autophagosomes was visualized by immunofluorescence analysis with LC3-II labelling. Results showed that Tiaozhi granule sera increase cell autophagic levels by increase of mRNA of Atg5, Atg7, Beclin-1, and mTOR and increase of autophagic flux and also number of autophagosomes. However, in response to rapamycin or Ang II stimulation, activated autophagic levels were alleviated by Tiaozhi granule sera by reduction of mRNA of Atg5, Atg7, Beclin-1, mTOR, autophagic flux, and also number of autophagosomes. Our present data demonstrate that Tiaozhi granule plays a dual role in response to different cell conditions, which is to increase cell autophagy under physiological condition and to suppress cell excessive autophagy under pathological condition.

## 1. Introduction

Traditional Chinese Medicine (TCM) has a long history and contributes to the prosperity of the Chinese nation. Yin-Yang theory and Five-Element theory are the ancient cosmology to explain the nature, materialism, and dialectics in China. TCM experts applied these theories to explain the changes of human body and to guide the diagnosis and treatment of patients. Consistent with the concept of homeostasis in modern medicine, human body maintains the balance of Yin-Yang: if it loses this balance, body gets sick. Herbs and other treatments are used to correct this imbalance of Yin-Yang [1, 2]. Tiaozhi granule is a composition of* Pollen Typhae Angustifoliae, Curcuma longa L.*, and* Rhizoma Alismatis* to treat hyperlipidemia (HLP) patients, which is developed by Wan Da-Cheng, a TCM grandmaster of Wu-Meng-Therapy. According to TCM theory, HLP in patients is caused by imbalance of blood stasis; thereafter, Tiaozhi granule aimed to rectify this imbalance. Three components of Tiaozhi granule have been widely applied in TCM for treatment of atherosclerosis, cancer, and inflammatory diseases [3–5]. Our recent study demonstrated that Tiaozhi granule could upregulate scavenger receptor class B type I (SR-BI) in hepatic cell [6], which may be one of its therapeutic mechanisms in controlling cholesterol homeostasis [7]. However, its beneficial effects on regulation of vascular homeostasis, especially on endothelial cell function, are still unknown.

The endothelium plays an important role in maintaining vascular homeostasis by its integrity and production of different relaxing and contractile factors. When pathological disturbance occurs in circulatory system, endothelial dysfunction will probably occur by acceleration of aging, increase of apoptosis, and reduction of regeneration [8, 9]. Autophagy is a highly conserved eukaryotic cellular recycling process, which plays an important role in regulating cell homeostasis via the degradation of cytoplasmic organelles, proteins, and macromolecules and the recycling of the breakdown products [10]. However, autophagy has also been demonstrated to serve as an alternative style of cell death [11]. Nowadays, autophagy is regarded as a two-edge sword, where mild induction of autophagy exerts cytoprotective effects, but massive induction of autophagy may cause excessive self-digestion of cell components and lead to cell death [11]. In the present study we hypothesized that Tiaozhi granule has dual effects on regulation of cell autophagy, which is upregulation of cell autophagy under physiological conditions, while suppressing excessive autophagy in response to pathological conditions, such as rapamycin or angiotensin II stimulation.

## 2. Materials and Methods

### 2.1. Serum Preparation for Cell Culture

Ten-week-old male Sprague-Dawley rats were purchased from Shanghai Laboratory Animal Center. Rats were housed under optimal conditions in the institutional animal facility. The experiments were performed according to the National Institutes of Health Guidelines for the Use of Laboratory Animals (NIH, publication number 85-23, revised 1996), which were approved by and performed according to guidelines for the care and use of animals established by Soochow University. Forty rats were randomly divided into 2 groups: (1) control group (n = 18); (2) Tiaozhi granule group (n = 22). Tiaozhi granule water solution or water was administrated by gastric feeding. The dosage applied in the present study is three times the clinical patient's dosage of Tiaozhi granule calculated by the ratio of surface area by human to rat. Three days after drugs administration (1 hour after third-day drug administration), rats were sacrificed under ether anesthetized condition and blood was obtained from abdominal aorta. Sera were further separated and collected in the same group and inactivated and then stored in - 80°C as our previous report [6].

### 2.2. Cell Culture

Human umbilical vein endothelial cells (HUVECs) were maintained in Dulbecco's modified Eagle's medium (DMEM) supplemented with 10% (v/v) fetal bovine serum.

Cells were cultured with different concentrations of sera (low, 1%; medium, 3%; high, 10%) obtained from the rats fed with Tiaozhi granule for 48 hours. HUVECs were also treated with rapamycin (10 mg/L) for 48 hours or with Ang II (10^−7^ mmol/L) for 24 hours under different concentration of Tiaozhi granule sera (low, 1%; medium, 3%; high, 10%). Additional sera from control rats were added to maintain 10% serum (v/v) for cell culture. Cells were harvested for further analysis.

### 2.3. Quantitative Analysis of the HUVECs Viability

Analysis of HUVECs viability was performed after cells were incubated with different concentrations of Tiaozhi granule sera (low, 1%; medium, 3%; high, 10%) for 48 hours or after cells were treated with rapamycin or Ang II under different concentrations of Tiaozhi granule sera condition (low, 1%; medium, 3%; high, 10%) for 48 hours as our previous report [12]. Briefly, MTT [3-[4,5-dimethylthiazol-2-yl]-2,5-diphenyl tetrazolium bromide] solution was added to the culture medium (final concentration was 5 mg/ml) 4 h before the end of treatment. The reaction was stopped by the addition of 10% acidified SDS (100 ul) to the cell culture. The absorbance value was measured at 570 nm using an automatic multiwell spectrophotometer (Bio-Rad, Richmond, CA, USA).

### 2.4. Real-Time PCR for Atg5, Atg7, Beclin-1, and mTOR mRNA Expression

mRNA expressions of Atg5, Atg7, Beclin-1, and mTOR were measured by RT-PCR as previously described [13]. In brief, total RNA was isolated from cells by guanidinium isothiocyanate-acid phenol extraction. One microgram of total RNA was used for reverse transcription and PCR assay. The primer pairs for each targeted genes are listed in Table 1. All mRNA expression was normalized to *β*-Actin mRNA.

### 2.5. Western Blot Assay for LC3, Beclin-1, and p62 Protein Expression

Expressions of LC3, Beclin-1, and p62 protein were measured by western blotting as our previous report [12]. Briefly, cells were harvested and lysates were resolved by 10% SDS-PAGE. Equal amounts of protein from each sample were resolved by 10% SDSPAGE. Proteins were transferred to PVDF membranes (Hybond TM-ECL; Amersham Pharmacia Biotech). The membranes were blocked for 2 h at room temperature with 5% skimmed milk in PBS and 0.1% Tween 20. The blots were incubated overnight with 1:1000 diluted primary antibodies: anti-LC3 (Abcam Corporation), Beclin-1 (Epitomics, Inc.), p62 (Abcam Corporation), and anti-GAPDH (Santa Cruz Biotech). Then the membranes were incubated for 1 hour with a secondary antibody (HRP-conjugated anti-rabbit Ig-G, 1:2000, Abgent). Excess antibody was washed off with TBS-T three times (15 minutes each) before incubation enhanced chemiluminescent reagent (ECL, R&D Systems Inc., Minneapolis, USA) for 1 min. Subsequently, the membrane was exposed to X-ray film. Immunoreactive bands were detected by the analysis of X-ray films using the software of Image J. The quantity of target proteins is normalized by GAPDH expression.

### 2.6. Immunofluorescence Detection of Autophagosomes in HUVECs

Detection of immunofluorescence of autophagosomes of LC3-II-associated membrane structures was carried out as shown in our previous report [12]. In brief, the HUVECs were fixed with 1:1 methanol and acetone, washed with PBS, and incubated in PBS containing 0.1% Triton X-100 for 10 min. After being washed with PBS again, the cells were then incubated for 1 h in a blocking solution of PBS containing 2% nonfat milk at room temperature. Cells were then incubated in blocking solution containing mouse anti-LC3-II (Abcam Corporation) overnight at 4°C, followed by incubation with Cy3-conjugated donkey anti-mouse immunoglobulin G antibody (Jackson Immuno Research Laboratories) with a dilution of 1:600 and fluorescein-isothiocyanate conjugated donkey anti-rabbit immunoglobulin G antibody (Jackson Immuno Research Laboratories) with a dilution of 1:800 for 2 h at room temperature. After rinsing with PBS, nuclei were stained with 40, 60-diamidino-2-phenylindole (DAPI; Sigma) for 10 min at room temperature. Finally, immunostained cells were rinsed with PBS and examined with a confocal microscope (C1 plus sci; Nikon). Microslips were randomly scanned with confocal microscope, and six microslips were used for each group.

### 2.7. Statistical Analysis

All data are presented as the mean ± SEM. Statistical significance between more than two groups was tested using one-way ANOVA followed by the Newman-Keel test.

*P* values < 0.05 are considered statistically significant.

## 3. Results

### 3.1. HUVECs Viability

**Figure 1:**
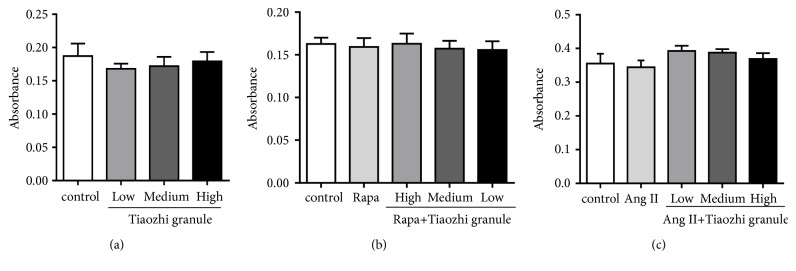
Effects of Tiaozhi granule on HUVECs viability. HUVECs were incubated with different concentration of Tiaozhi granule sera for 48 hours (a) or were treated with rapamycin (Rapa) for 48 hours (b) or Ang II for 24 hours (c) under different concentration of Tiaozhi granule sera. Cell viability was analyzed by MTT assay. Data from each group is expressed as absorbance of each well at 570 nm and is presented as mean ± SEM (n = 8).

HUVECs viability was examined using MTT assay. Results showed that Tiaozhi granule sera under three different concentrations (low, 1%; medium, 3%; high, 10%, 48 hours) did not affect cell viability (Figure 1(a)).

Rapamycin (10 mg/L, 48 hours) or Ang II (10^−7^ mmol/L, 24 hours) also had no marked effect on HUVECs viability (Figures 1(b) and 1(c)). Meanwhile, cell viability was also not affected by rapamycin (10 mg/L, 48 hours) or Ang II treatment (10^−7^ mmol/L, 24 hours) under different concentrations of Tiaozhi granule sera (low, 1%; medium, 3%; high, 10%) incubation (Figures 1(b) and 1(c)). These data revealed that Tiaozhi granule does not affect cell viability under both physiological or pathological conditions.

### 3.2. Effect of Tiaozhi Granule on HUVECs Autophagy Levels

**Figure 2:**
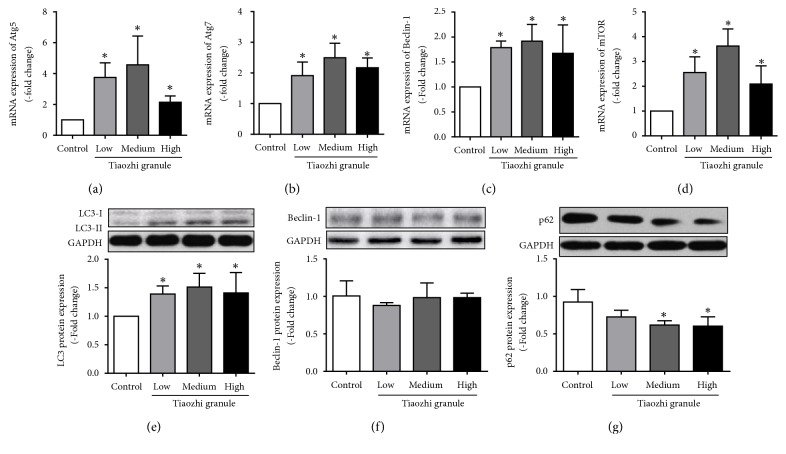
Effects of Tiaozhi granule on autophagy level in HUVECs. (a) Effect of Tiaozhi granule on Atg5 mRNA expression (n = 6). (b) Effect of Tiaozhi granule on Atg7 mRNA expression (n = 6). (c) Effect of Tiaozhi granule on Beclin-1 mRNA expression (n = 6). (d) Effect of Tiaozhi granule on mTOR mRNA expression (n = 6). (e) Effect of Tiaozhi granule on LC3 protein expression (n = 6). Upper part is representative blot of LC3 and GAPDH; lower part is the densitometric analysis of the ratio of LC3-II to LC3-I normalized to GAPDH. (f) Effect of Tiaozhi granule on Beclin-1 protein expression (n = 6). Upper part is representative blot of Beclin-1 and GAPDH; lower part is the densitometric analysis of Beclin-1 expression normalized to GAPDH. (g) Effect of Tiaozhi granule on p62 protein expression (n = 6). Upper part is representative blot of p62 and GAPDH; lower part is the densitometric analysis of the ratio of p62 expression normalized to GAPDH. Data were presented as mean ± SEM. *∗*  *P* < 0.05 compared with control group.

mRNA expressions of autophagy related genes were analyzed. Results showed that Tiaozhi granule sera could significantly increase mRNA expression of Atg5, Atg7, Beclin-1, and mTOR under different concentrations (Figures 2(a)–2(d)).

Autophagy flux proteins were also detected. Data demonstrated that ratio of LC3II to LC3-I was markedly increased under different concentration of Tiaozhi granule sera incubation (Figure 2(e)); p62 protein expression was dose-dependently reduced under different concentration of Tiaozhi granule sera incubation (Figure 2(g)). However, Beclin-1 protein expression was not increased under different concentration of Tiaozhi granule sera incubation (Figure 2(f)).

**Figure 3:**
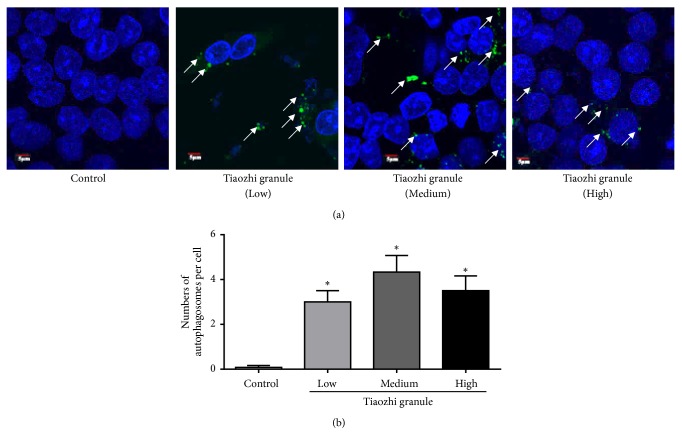
Immunofluorescent analysis of Tiaozhi granule on the formation of autophagosomes. (a) Representative images of immunofluorescent detection of LC3-II. Nucleus was stained with DAPI in blue color. Autophagosomes were observed with LC3-II protein expression. Green dots in the cell cytosol indicate autophagosomes (marked with white arrows). Magnification of the image is 400X. (b) Numbers of autophagosomes in HUVECs within each treatment group were counted. Data are presented as mean ± SEM (n = 8). *∗p* <0.05 versus control.

In addition, formation of autophagosome was observed. Results showed that Tiaozhi granule sera under different concentrations could significantly increase the formation of autophagosome (Figure 3(a)). The number of autophagosomes under different concentrations of Tiaozhi granule sera incubation was markedly increased (Figure 3(b)). These results revealed that Tiaozhi granule could upregulate cell autophagy levels under physiological conditions.

### 3.3. Effects of Tiaozhi Granule on Rapamycin Activated Cell Autophagy

**Figure 4:**
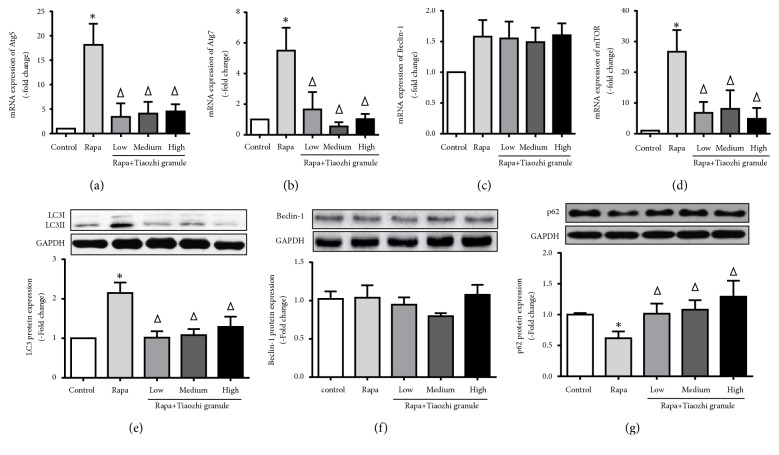
Effects of Tiaozhi granule on rapamycin activated autophagy in HUVECs. (a) Effect of Tiaozhi granule on rapamycin induced Atg5 mRNA expression (n = 6). (b) Effect of Tiaozhi granule on rapamycin induced Atg7 mRNA expression (n = 6). (c) Effect of Tiaozhi granule on rapamycin induced Beclin-1 mRNA expression (n = 6). (d) Effect of Tiaozhi granule on rapamycin induced mTOR mRNA expression (n = 6). (e) Effect of Tiaozhi granule on rapamycin induced LC3 protein cleavage (n = 6). Upper part is representative blot of LC3 and GAPDH; lower part is the densitometric analysis of the ratio of LC3-II to LC3-I normalized to GAPDH. (f) Effect of Tiaozhi granule on rapamycin induced Beclin-1 protein expression (n = 6). Upper part is representative blot of Beclin-1 and GAPDH; lower part is the densitometric analysis of Beclin-1 expression normalized to GAPDH. (g) Effect of Tiaozhi granule on rapamycin induced p62 protein degradation (n = 6). Upper part is representative blot of p62 and GAPDH; lower part is the densitometric analysis of the ratio of p62 expression normalized to GAPDH. Data were presented as mean ± SEM. *∗*  *P* < 0.05 compared with control group. Δ  *P* < 0.05 compared with rapamycin group.

Rapamycin, an inhibitor of mTOR, is a well-known cell autophagy activator. Results showed that rapamycin significantly increased mRNA expression of Atg5, Atg7, and mTOR and slightly increased Beclin-1 mRNA expression (Figures 4(a)–4(d)). Interestingly, increased mRNA expressions of Atg5, Atg7, and mTOR were markedly suppressed under Tiaozhi granule sera incubation with different concentrations (Figures 4(a), 4(b), and 4(d)); slightly increased Beclin-1 mRNA expression was not affected by Tiaozhi granule sera under any concentrations (Figure 4(c)).

Similarly, autophagy flux proteins were analyzed. Rapamycin significantly increased autophagy flux by elevation of LC3 cleavage (Figure 4(e)) and degradation of p62 (Figure 4(g)) but not expression of Beclin-1 (Figure 4(g)). Tiaozhi granule sera could inhibit rapamycin induced increase of autophagy flux by reducing LC3 cleavage (Figure 4(e)) and degradation of p62 (Figure 4(g)), and it also had no effect on Beclin-1 expression (Figure 4(f)).

**Figure 5:**
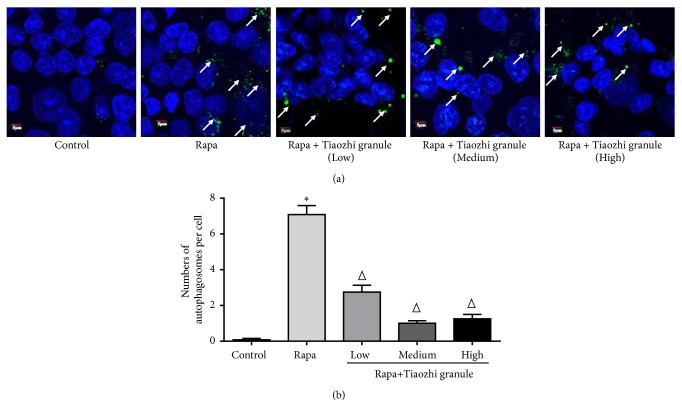
Immunofluorescent analysis of Tiaozhi granule on rapamycin induced formation of autophagosomes. (a) Representative images of immunofluorescent detection of LC3-II. Nucleus was stained with DAPI in blue color. Autophagosomes were observed with LC3-II protein expression. Green dots in the cell cytosol indicate autophagosomes (marked with white arrows). Magnification of the image is 400X. (b) Numbers of autophagosomes in HUVECs within each treatment group were counted. Data are presented as mean ± SEM (n = 8). *∗p* <0.05 versus control. Δ*P* < 0.05 compared with rapamycin group.

In order to confirm the effects of Tiaozhi granule on rapamycin activated autophagy levels, formation of autophagosome was also observed. Data showed that rapamycin treatment significantly increased formation of autophagosome (Figures 5(a) and 5(b)). Under different concentrations of Tiaozhi granule sera incubation, increased formation of autophagosome was markedly reduced (Figures 5(a) and 5(b)). These results demonstrated that Tiaozhi granule could alleviate rapamycin induced cell autophagy.

### 3.4. Effects of Tiaozhi Granule on Ang II-Induced Cell Autophagy

**Figure 6:**
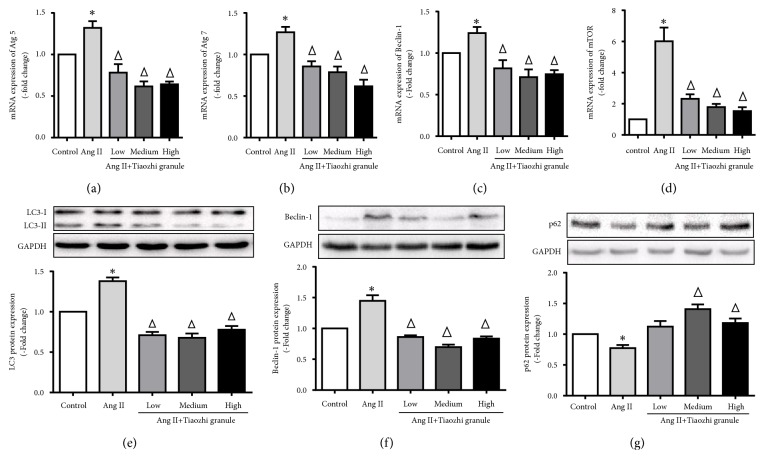
Effects of Tiaozhi granule on Ang II-induced autophagy in HUVECs. (a) Effect of Tiaozhi granule on Ang II-induced Atg5 mRNA expression (n = 6). (b) Effect of Tiaozhi granule on Ang II-induced Atg7 mRNA expression (n = 6). (c) Effect of Tiaozhi granule on Ang II-induced Beclin-1 mRNA expression (n = 6). (d) Effect of Tiaozhi granule on Ang II-induced mTOR mRNA expression (n = 6). (e) Effect of Tiaozhi granule on Ang II-induced LC3 protein cleavage (n = 6). Upper part is representative blot of LC3 and GAPDH; lower part is the densitometric analysis of the ratio of LC3-II to LC3-I normalized to GAPDH. (f) Effect of Tiaozhi granule on Ang II-induced Beclin-1 protein expression (n = 6). Upper part is representative blot of Beclin-1 and GAPDH; lower part is the densitometric analysis of Beclin-1 expression normalized to GAPDH. (g) Effect of Tiaozhi granule on Ang II-induced p62 protein degradation (n = 6). Upper part is representative blot of p62 and GAPDH; lower part is the densitometric analysis of the ratio of p62 expression normalized to GAPDH. Data were presented as mean ± SEM. *∗*  *P* < 0.05 compared with control group. Δ  *P* < 0.05 compared with rapamycin group.

Ang II, a growth factor, has multiple effects on endothelial cells and also has been demonstrated to activate cell autophagy. Data showed that Ang II significantly increased mRNA expression of Atg5, Atg7, Beclin-1, and mTOR (Figures 6(a)–6(d)). Also, increased mRNA expressions of Atg5, Atg7, Beclin-1, and mTOR were markedly suppressed under Tiaozhi granule sera incubation with different concentrations (Figures 4(a)–4(d)).

Ang II also significantly increased autophagy flux by elevation of LC3 cleavage (Figure 6(e)), increase of Beclin-1 expression (Figure 6(f)), and degradation of p62 (Figure 6(g)). Tiaozhi granule sera could inhibit Ang II-induced increase of autophagy flux by reducing LC3 cleavage (Figure 6(e)), suppressing Beclin-1 expression (Figure 6(f)), and degradation of p62 (Figure 4(g)).

**Figure 7:**
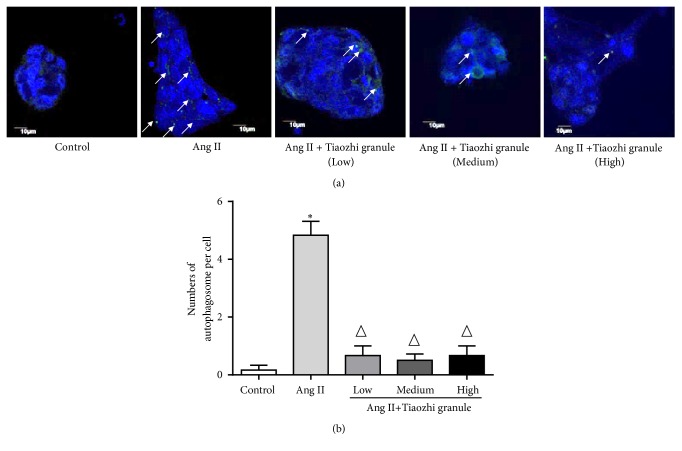
Immunofluorescent analysis of Tiaozhi granule on Ang II-induced formation of autophagosomes. (a) Representative images of immunofluorescent detection of LC3-II. Nucleus was stained with DAPI in blue color. Autophagosomes were observed with LC3-II protein expression. Green dots in the cell cytosol indicate autophagosomes (marked with white arrows). Magnification of the image is 400X. (b) Numbers of autophagosomes in HUVECs within each treatment group were counted. Data are presented as mean ± SEM (n = 8). *∗p* <0.05 versus control. Δ*P* < 0.05 compared with rapamycin group.

In order to confirm the effects of Tiaozhi granule on Ang II-induced autophagy levels, formation of autophagosome was also observed. Data showed that Ang II stimulation significantly increased formation of autophagosome (Figures 7(a) and 7(b)). Under different concentrations of Tiaozhi granule sera incubation, increased formation of autophagosome was markedly reduced (Figures 7(a) and 7(b)). These results demonstrated that Tiaozhi granule could alleviate Ang II-induced cell autophagy.

## 4. Discussion

In the present study, we demonstrated that Tiaozhi granule could upregulate cell autophagy levels under physiological conditions and could suppress rapamycin or Ang II activated cell autophagy levels, which exerts dual-direction regulation effects consistent with TCM Yin-Yang balance theory.

One component of Tiaozhi granule is* Pollen Typhae Angustifoliae*, from which flavonoids and polysaccharides have been extracted, and those extractions act as main effective chemicals [14]. Previous basic studies have demonstrated the effects of* Pollen Typhae Angustifoliae* on immune system [15], on coagulation system [16], and on metabolism [17]. In addition, anti-inflammatory activity and antioxidant potential of* Pollen Typhae Angustifoliae* were also reported [3, 18]. Another component of Tiaozhi granule is* Curcuma longa L.*, which mainly has been used as an antihepatotoxic drug [4, 19, 20]. Existing literatures have demonstrated that* Curcuma longa L.* possesses liver protection effects [21–23]. The third component of Tiaozhi granule is* Rhizoma Alismatis*, which also has been demonstrated to exert antihyperlipidemic and antiatherosclerotic effects [5, 24]. Recently, we compared the effects of three components on regulation of SR-BI expression and demonstrated that* Pollen Typhae Angustifoliae* is important for the regulatory effect coordinating with* Curcuma longa L. and Rhizoma Alismatis* [6]. Based on these observations, we can conclude that each component of Tiaozhi granule could improve blood circulation or related systems. Thereafter, combination of these components to ameliorate the imbalance of blood circulation system is reasonable, which is consistent with the concept of Yin-Yang in TCM.

Autophagy is a lysosome-mediated degradation process for nonessential or damaged cellular constituents, which is primarily a survival mechanism in response to stress such as starvation. Autophagy has been regarded as a process of cytosolic renovation and cell makeover rather than cell death in the mammalian development [25]. However, autophagy acts as a prodeath pathway which has also been widely reviewed [11]. According to TCM Yin-Yang theory, the discrepancy may be explained by the imbalance of cell Yin-Yang, which is high Yin (low autophagy) or high Yang (high autophagy) will result in cell death, while maintaining suitable Yin-Yang (autophagy) probably is beneficial for cell survival. Thereafter, the key point is to properly regulate the cell autophagy levels. Recently, active compounds from TCM have been found to modulate the levels of autophagy in tumor cells, nerve cells, myocardial cells, and endothelial cells [26]. In Tiaozhi granule components, flavonoid, extractor of* Pollen Typhae Angustifoliae*, has been demonstrated to induce autophagy via ROS-dependent mitochondrial dysfunction and loss of ATP involving BNIP3 in human MCF7 breast cancer cells [27]. Another extractor of* Pollen Typhae Angustifoliae*, polysaccharide, also has been reported to induce apoptosis and autophagy in human hepatoma SMMC-7721 cells [28]. Ethanol extract of* alismatis rhizome* inhibits adipocyte autophagy of OP9 cells treatment with adipogenic inducers [29]. Curcumin, the principal* Curcuma longa L.*, activates autophagy and attenuates oxidative damage in EA.hy926 cells via the Akt/mTOR pathway [30]. These observations support the fact that components of Tiaozhi granule could regulate cell autophagy levels; however, the effects of Tiaozhi granule on regulation of vascular cells are still unclear. Due to important role of endothelium in circulatory system, we firstly focused on the effects of Tiaozhi granule on regulation of autophagy levels in endothelial cells.

In the present study, firstly our results clearly show that even under highest concentrations of Tiaozhi granule sera treatment cell viability is not affected, suggesting the safety of Tiaozhi granule applied in the present study design (Figure 1). Secondly, our results also show that Tiaozhi granule sera treatment could increase autophagy related regulator gene mRNA expression such as Atg5, Atg7, Beclin-1, and mTOR, accompanied with increase of protein expression of LC-3 II and decrease of p62 protein expression (Figure 2). These results suggest that Tiaozhi granule could improve cell autophagy levels under physiological conditions (high Yin condition), confirmed by autophagosome observation, indicating that Tiaozhi granule could enhance cell self-clearance to improve cell function without affecting cell viability. Thirdly, in response to rapamycin or Ang II stimulation conditions (high Yang condition), Tiaozhi granule effectively suppressed rapamycin induced increase of Atg5, Atg7, and mTOR mRNA expression and inhibited rapamycin induced increase of LC3II and decrease of p62 protein expression; similarly, Tiaozhi granule also markedly suppressed Ang II-induced increase of Atg5, Atg7, Beclin-1, and mTOR mRNA expression and reversed Ang II-induced increase of LC3-II and Beclin-1 and decrease of p62 protein expression. These results clearly demonstrated that Tiaozhi granule could exert inhibitory effects in response to increased cell autophagy levels. Our present results also revealed a novel mechanism of Tiaozhi granule in regulation of cell function, which is concordance with TCM treatment principle to adjust imbalance of Yin-Yang.

It should be noted that cell viability is not markedly affected under present treatments with rapamycin or Ang II, although these two treatments have been widely reported to be related to cell death or survival. Our previous study also observed that under present condition of treatment Ang II did not affect cell viability [12]. In the present study, we found that under physiological condition Tiaozhi granule could increase Beclin-1 mRNA expression, but not protein expression; also, our results showed that rapamycin did not affect Beclin-1 expression and Tiaozhi granule could not increase Beclin-1 under rapamycin treatment; these discrepancies are still unknown. Detailed mechanism of how Tiaozhi granule corrects imbalance of cell condition is unknown; furthermore, role of each component of Tiaozhi granule in regulation of cell autophagy is also unknown; further investigations are still needed.

In conclusion, our present study demonstrates a novel regulatory mechanism of Tiaozhi granule in cell autophagy, which is improving cell autophagy under physiological condition and suppressing cell autophagy under pathological conditions.

## Figures and Tables

**Table 1:** Primer pairs of cDNA for each targeted gene.

Gene	Sequence	bp
Beclin-1	Sense 5′ CCAGATGCGTTATGCCCAGAC 3′ Anti-sense 5′ CATTCCATTCCACGGGAACAC 3′	149

mTOR	Sense 5′ AAACTGCACGTCAGCACCATC 3′ Anti-sense 5′ AGCCGTCTCAGCCATTCCA 3′	192

ATG5	Sense 5′CCATCAATCGGAAACTCATGGA 3′ Anti-sense 5′ATCTGCAGCCACAGGACGAA 3′	128

ATG7	Sense 5′ CTGTAACTTAGCCCAGTACCCTGGA 3′ Anti-sense 5′TACGGTCACGGAAGCAAACAAC 3′	113

Actin	Sense 5′TGGCACCCAGCACAATGAA 3′ Anti-sense 5′CTAAGTCATAGTCCGCCTAGAAGCA 3′	128

## Data Availability

The data used to support the findings of this study are included within the article.

## References

[1] Wei-Bo L. (1983). The modern scientific research on the theory of traditional chinese medicine. *Ancient Science of Life*.

[2] Cheng J. T. (2000). Review: drug therapy in Chinese traditional medicine. *Clinical Pharmacology and Therapeutics*.

[3] Varpe S. S., Juvekar A. R., Bidikar M. P., Juvekar P. R. (2012). Evaluation of anti-inflammatory activity of *Typha angustifolia* pollen grains extracts in experimental animals. *Indian Journal of Pharmacology*.

[4] Selvam R., Subramanian L., Gayathri R., Angayarkanni N. (1995). The anti-oxidant activity of turmeric (*Curcuma longa*). *Journal of Ethnopharmacology*.

[5] Tian T., Chen H., Zhao Y.-Y. (2014). Traditional uses, phytochemistry, pharmacology, toxicology and quality control of Alisma orientale (Sam.) Juzep: a review. *Journal of Ethnopharmacology*.

[6] Yu X., Zhao X.-D., Bao R.-Q., Yu J.-Y., Zhang G.-X., Chen J.-W. (2016). Effects of extracts from tiaozhi granule and its components on expression of scavenger receptor class B type I. *Evidence-Based Complementary and Alternative Medicine*.

[7] Zanoni P., Khetarpal S. A., Larach D. B. (2016). Rare variant in scavenger receptor BI raises HDL cholesterol and increases risk of coronary heart disease. *Science*.

[8] Tang E. H., Vanhoutte P. M. (2010). Endothelial dysfunction: a strategic target in the treatment of hypertension?. *Pflügers Archiv: European Journal of Physiology*.

[9] Becher U. M., Endtmann C., Tiyerili V., Nickenig G., Werner N. (2011). Endothelial damage and regeneration: The role of the renin-angiotensin- aldosterone system. *Current Hypertension Reports*.

[10] Parzych K. R., Klionsky D. J. (2014). An overview of autophagy: morphology, mechanism, and regulation. *Antioxidants & Redox Signaling*.

[11] Denton D., Xu T., Kumar S. (2015). Autophagy as a pro-death pathway. *Immunology & Cell Biology*.

[12] Yu K.-Y., Wang Y.-P., Wang L.-H. (2014). Mitochondrial KATP channel involvement in angiotensin II-induced autophagy in vascular smooth muscle cells. *Basic Research in Cardiology*.

[13] Jiang Y., Tian L. L., Wang L. H. (2017). Cardioprotective effects of Serca2a overexpression against ischemiareperfusion- induced injuries in rats. *Current Gene Therapy*.

[14] Xi X. R., Li S. X. (2000). Analysis on contents of flavonoids and polysaccharides in pollen of Typha angustifolia L. and its different processed products. *Zhongguo Zhong Yao Za Zhi*.

[15] Qin F., Sun H.-X. (2005). Immunosuppressive activity of Pollen Typhae ethanol extract on the immune responses in mice. *Journal of Ethnopharmacology*.

[16] Ohkura N., Tamura K., Tanaka A., Matsuda J., Atsumi G.-I. (2011). Experimental study on the hemostatc activity of Pollen Typhae: a traditional folk medicine used by external and oral application. *Blood Coagulation & Fibrinolysis*.

[17] Feng X.-T., Chen Q., Xie Z. (2014). Pollen Typhae total flavone improves insulin resistance in high-fat diet and low-dose streptozotocin-induced type 2 diabetic rats. *Bioscience, Biotechnology, and Biochemistry*.

[18] Sardar A. A., Akhan Z. U., Perveen A., Farid S., Khan I. U. (2014). In vitro antioxidant potential and free radical scavenging activity of various extracts of pollen of Typha domigensis Pers. *Pakistan Journal of Pharmaceutical Sciences*.

[19] Aggarwal B. B., Sundaram C., Malani N., Ichikawa H. (2007). Curcumin: the Indian solid gold. *Advances in Experimental Medicine and Biology*.

[20] Corson T. W., Crews C. M. (2007). Molecular understanding and modern application of traditional medicines: triumphs and trials. *Cell*.

[21] Tu C.-T., Yao Q.-Y., Xu B.-L., Wang J.-Y., Zhou C.-H., Zhang S.-C. (2012). Protective effects of curcumin against hepatic fibrosis induced by carbon tetrachloride: modulation of high-mobility group box 1, Toll-like receptor 4 and 2 expression. *Food and Chemical Toxicology*.

[22] Lee H. Y., Kim S. W., Lee G. H. (2016). Turmeric extract and its active compound, curcumin, protect against chronic CCl4-induced liver damage by enhancing antioxidation. *BMC Complementary and Alternative Medicine*.

[23] Xu J., Fu Y., Chen A. (2003). Activation of peroxisome proliferator-activated receptor-*γ* contributes to the inhibitory effects of curcumin on rat hepatic stellate cell growth. *American Journal of Physiology—Gastrointestinal and Liver Physiology*.

[24] Li S., Jin S., Song C. (2016). The metabolic change of serum lysophosphatidylcholines involved in the lipid lowering effect of triterpenes from *Alismatis rhizoma* on high-fat diet induced hyperlipidemia mice. *Journal of Ethnopharmacology*.

[25] Cecconi F., Levine B. (2008). The role of autophagy in mammalian development: cell makeover rather than cell death. *Developmental Cell*.

[26] Huang X.-P., Ding H., Lu J.-D., Tang Y.-H., Deng B.-X., Deng C.-Q. (2015). Autophagy in cerebral ischemia and the effects of traditional Chinese medicine. *Journal of Integrative Medicine*.

[27] Jiang K., Wang W., Jin X., Wang Z., Ji Z., Meng G. (2015). Silibinin, a natural flavonoid, induces autophagy via ROS-dependent mitochondrial dysfunction and loss of ATP involving BNIP3 in human MCF7 breast cancer cells. *Oncology Reports*.

[28] Zhao F., Zhao J., Song L., Zhang Y., Guo Z., Yang K. (2017). The induction of apoptosis and autophagy in human hepatoma SMMC-7721 cells by combined treatment with vitamin C and polysaccharides extracted from Grifola frondosa. *Apoptosis*.

[29] Park Y. J., Kim M. S., Kim H. R. (2014). Ethanol extract of *Alismatis rhizome* inhibits adipocyte differentiation of OP9 cells. *Evidence-Based Complementary and Alternative Medicine*.

[30] Guo S., Long M., Li X., Zhu S., Zhang M., Yang Z. (2016). Curcumin activates autophagy and attenuates oxidative damage in EA.hy926 cells via the Akt/mTOR pathway. *Molecular Medicine Reports*.

